# Programming Hydrogel Release Kinetics to Tissue Healing Phases: From Network Design to Therapeutic Synchronization

**DOI:** 10.3390/gels12090805

**Published:** 2026-09-03

**Authors:** Qiao Chen, Tong Wang, Lusi Zou, Qi Dong

**Affiliations:** 1 School of Pharmacy, Hubei University of Chinese Medicine, Wuhan 430065, China; qchen0305@hbucm.edu.cn (Q.C.); 25326000914@stmail.hbucm.edu.cn (T.W.); 2 College of Chemistry and Chemical Engineering, Hubei University, Wuhan 430062, China; 3 School of Biomedical Engineering and Health, Wuhan Textile University, Wuhan 430200, China; 201910301001@whu.edu.cn

**Keywords:** hydrogel, drug delivery, release kinetics, wound healing, tissue regeneration, stimuli-responsive, sequential release, phase-matched therapy, chronotherapy, regenerative medicine

## Abstract

The sequential phases of tissue healing—inflammation, proliferation, and remodeling—demand distinct pharmacokinetic profiles that conventional drug delivery systems fail to provide, creating a “chronotherapy gap” that contributes to chronic wound pathologies. Hydrogels, with their highly tunable network structures, offer a unique platform to program release kinetics in synchrony with these healing timelines. This review systematically examines design strategies for phase-synchronized hydrogel systems, categorized into three hierarchical paradigms: intrinsic network control (crosslinking density, degradation kinetics, and architectural engineering) that pre-programs release profiles; extrinsic/responsive control (endogenous pH/ROS/MMP/glucose and exogenous NIR/ultrasound/electro/magnetic triggers) that enables on-demand phase-shifting; and integrated systems that combine passive spatial compartmentalization with active responsiveness. We survey representative applications across cutaneous wounds, bone defects, cartilage, tendon, myocardial, and neural tissues, highlighting both common design principles and tissue-specific adaptations. Key translational bottlenecks—including in vivo–in vitro discrepancies, cargo stability, sterilization challenges, and regulatory complexity—are critically examined, alongside emerging frontiers such as closed-loop biosensing, artificial intelligence-driven design, and four-dimensional printing. We conclude that the field is evolving from passive drug depots toward active therapeutic synchronizers, where material programming is set to the body’s biological clock, offering a transformative paradigm for regenerative medicine.

## 1. Introduction

Tissue repair following injury is a highly orchestrated and dynamic biological process, classically divided into three overlapping but temporally distinct phases: inflammation, proliferation, and remodeling [1]. During the inflammatory phase (0–3 days post-injury), the wound site is characterized by neutrophil and macrophage infiltration, a burst of reactive oxygen species (ROS), and elevated levels of pro-inflammatory cytokines such as TNF-α and IL-1β, which collectively serve to eliminate pathogens and clear cellular debris [2,3]. The proliferative phase (3–14 days) involves the migration and proliferation of fibroblasts and endothelial cells, the formation of granulation tissue, and the onset of angiogenesis driven by growth factors including vascular endothelial growth factor (VEGF) and platelet-derived growth factor (PDGF) [4,5]. Finally, the remodeling phase (>14 days) is marked by collagen cross-linking and re-organization, the apoptosis of myofibroblasts, and the gradual maturation of the extracellular matrix (ECM) under the regulation of matrix metalloproteinases (MMPs) [6,7]. The successful progression through these phases—and the timely transition between them—is essential for functional tissue restoration. Conversely, dysregulation at any stage, particularly the failure to resolve inflammation and transition to proliferation, is a hallmark of chronic, non-healing wounds such as diabetic ulcers [8].

Despite significant advances in wound care, conventional therapeutic strategies—including systemic antibiotic administration, topical antimicrobial creams, and passive occlusive dressings—often fail to achieve satisfactory clinical outcomes. A principal limitation of these approaches lies in their inability to temporally synchronize drug delivery with the evolving pathophysiology of the healing wound [1]. Systemic administration dilutes therapeutic agents before they reach the target site, while conventional topical formulations typically provide a single, uncontrolled bolus release that neither matches the acute demands of the inflammatory phase nor sustains the protracted signaling required for proliferation and remodeling [9]. As a result, therapeutic concentrations may be insufficient during critical windows, or conversely, excessive and prolonged exposure may interfere with later-stage healing processes, such as angiogenesis and ECM remodeling. This “chronotherapy gap”—the mismatch between drug availability and biological need—represents a fundamental obstacle in wound management [1,10].

Hydrogels, three-dimensional crosslinked polymer networks with high water content, have emerged as exceptionally versatile platforms for localized drug delivery in regenerative medicine [11]. Their inherent biocompatibility, tunable physicochemical properties, and ability to create a moist wound environment conducive to healing make them ideal candidates for wound dressing applications [12,13]. More importantly, hydrogels offer a unique capacity for precise control over drug release kinetics through rational network design. By modulating crosslinking density, polymer composition, network architecture (e.g., bilayer, coreshell, or graded structures), and incorporating stimuli-responsive moieties, researchers can program hydrogels to exhibit distinct release profiles—ranging from rapid burst release to zero-order sustained release to degradation-controlled erosion [14,15]. This design flexibility has enabled the development of “smart” hydrogel systems capable of responding to endogenous microenvironmental cues such as pH, ROS, and MMPs, or exogenous triggers including temperature, light, and ultrasound [16,17]. Such responsive systems hold the potential to deliver therapeutic agents precisely when and where they are needed, in synchrony with the changing demands of the healing cascade [18].

In recent years, a growing body of research has focused on tailoring hydrogel release kinetics to the specific temporal requirements of different healing phases. These phase-synchronized delivery systems aim to achieve sequential release of multiple bioactive agents—for example, rapid antimicrobial delivery during early inflammation, followed by sustained angiogenic factor release during proliferation, and ultimately anti-fibrotic agents during remodeling [19,20]. Such programmed release strategies represent a paradigm shift from passive wound coverage to active therapeutic orchestration [21]. While these studies have demonstrated the feasibility of phase-synchronized delivery systems, the design principles governing such systems—spanning from passive network control to stimuli-responsive activation and their integration—have yet to be systematically synthesized across different tissue types. Two recent reviews have provided valuable frameworks—one for sequential-release hydrogels [22] and another for bone regeneration [23]. However, a comprehensive review that bridges these design strategies with the distinct healing timelines of multiple soft and hard tissues remains lacking, particularly one that provides a cross-tissue comparative perspective.

Unlike previous reviews that focus on single tissues or isolated design strategies, this review systematically categorizes hydrogel programming into passive, active, and integrated paradigms and provides a cross-tissue comparative analysis. This three-tier classification—intrinsic (passive), extrinsic (active), and integrated (synergistic) control—offers a rational design roadmap for phase-matched hydrogel development. Passive control serves as the baseline strategy for applications where healing timelines are predictable and reproducible, such as clean surgical wounds. Active control is indicated when the healing microenvironment is highly variable and on-demand adaptation is required, as in infected or diabetic wounds. Integrated control becomes necessary when neither passive nor active strategies alone can achieve the complex, multi-stage release profiles demanded by chronic or severe tissue injuries. Building on this framework, this review provides a systematic overview of recent advances in tailoring hydrogel release kinetics to align with the sequential phases of tissue healing. We first summarize the biological demands of each healing phase and their pharmacokinetic implications. We then examine the network design strategies—including crosslinking chemistry, architectural engineering, and composite fabrication—that enable programmable release profiles. Next, we discuss microenvironment-responsive trigger mechanisms that facilitate on-demand phase shifting. Finally, we survey representative applications across six tissue types—cutaneous wounds, bone defects, cartilage, tendon, myocardial, and neural tissues—and offer perspectives on the challenges and future directions for translating these intelligent systems into clinical practice.

**Figure 1:**
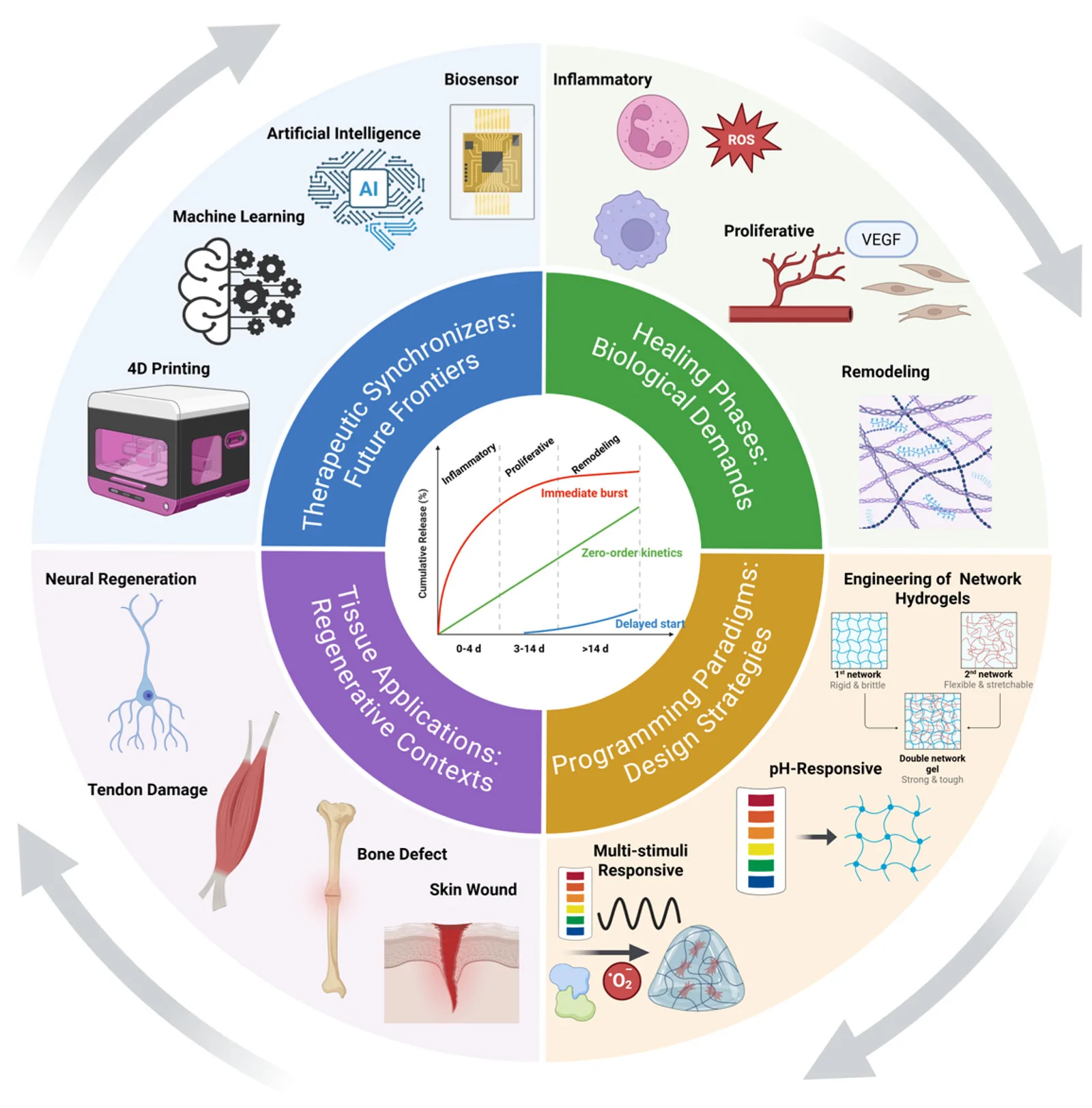
Graphical abstract of phase-synchronized hydrogel design. This schematic illustrates the closed-loop logic of the review: tissue healing phases (inflammation, proliferation, remodeling) define distinct release requirements (burst, sustained, ultra-sustained/delayed). These are addressed through hierarchical hydrogel programming paradigms—passive, active, and integrated control—and validated across tissue applications (skin, bone, cartilage/tendon, myocardial/neural). The field ultimately evolves toward active therapeutic synchronizers, where material programming matches the body’s biological clock. Circular arrows denote feedback between biological demands and materials design. The central panel shows release kinetic profiles matched to each phase. Created in BioRender. Chen, Q. (2026) https://BioRender.com/bog98a5.

The conceptual framework of this review—connecting healing phases, release kinetics, design strategies, and tissue applications—is graphically summarized in Figure 1.

## 2. Healing Phase-Specific Requirements for Hydrogel Release Kinetics

The previous section established the phasic nature of wound healing. To translate this biological timeline into tangible design criteria, this section systematically extracts the release specifications that hydrogels must satisfy at each stage. The temporal intervals presented here—inflammation (0–4 days), proliferation (3–14 days), and remodeling (>14 days)—are primarily derived from cutaneous wound healing models [24], which provide the most extensively characterized timeline for tissue repair. However, these durations are not universally applicable across all tissues. Bone healing, for instance, extends over weeks to months [24]; myocardial regeneration involves distinct inflammatory and reparative phases that overlap differently; and tendon healing follows a prolonged time course [25,26]. Tissue-specific deviations are addressed in Section 4, where the design strategies are contextualized to each application. The pharmacokinetic specifications outlined here should therefore be understood as a reference framework rather than fixed temporal boundaries. For each phase, we identify the core pharmacokinetic requirement—whether burst release, sustained zero-order delivery, or ultra-sustained and delayed release—and briefly indicate the material characteristics needed to meet it. This “design specification” approach provides the biological foundation for the engineering strategies detailed in Section 3. Importantly, the discussion here focuses on what release profile is needed and why; how to achieve it is the subject of the following section.

### 2.1. Inflammatory Phase: The Burst-Release Mandate

The inflammatory phase spans the first 0–4 days post-injury and is characterized by neutrophil infiltration, macrophage activation, and a burst of reactive oxygen species (ROS) that together eliminate pathogens and clear debris [2]. The wound microenvironment during this window exhibits elevated ROS, acidic pH (approximately 5.5–6.5) [16,27], and high concentrations of pro-inflammatory cytokines [3].

The pharmacokinetic specification for hydrogel design during the inflammatory phase depends critically on the wound context. For contaminated or infected wounds, delivery must achieve rapid, high-concentration release within the first 24–48 h to eradicate bacterial contamination before biofilm establishment [28]. This ‘burst release’ profile is essential in infectious contexts, where delayed antimicrobial intervention is a primary driver of chronic wound development, as persistent M1 macrophage dominance prevents the transition to the proliferative phase [29,30]. However, for sterile injuries in tissues such as bone, tendon, myocardium, or neural tissue, the inflammatory phase primarily involves the clearance of debris and the initiation of reparative signaling; the release of immunomodulatory agents—rather than antimicrobials—may be more relevant, and the requirement for a rapid burst is less stringent. Promoting the M1-to-M2 macrophage transition is critical, as persistent M1 dominance is a hallmark of non-healing wounds [18,29,31].

### 2.2. Proliferative Phase: The Sustained-Release Mandate

The proliferative phase spans days 3–14 and involves granulation tissue formation, angiogenesis, fibroblast proliferation, and re-epithelialization [4]. This stage is driven by a cascade of growth factors—VEGF, PDGF, FGF, and TGF-β—each with distinct temporal expression peaks that collectively orchestrate tissue reconstruction [5,32,33].

The pharmacokinetic specification for hydrogel design shifts from burst release to sustained, near-zero-order release over 7–14 days. Growth factors have short half-lives, making a single bolus physiologically inadequate [11,17]. Importantly, the sequence of growth factor presentation matters: effective angiogenesis requires VEGF first (endothelial recruitment), followed by FGF-2 (proliferation/migration), and PDGF (vessel stabilization) [32]. This temporal dependency indicates that sequential release of multiple growth factors, rather than simultaneous co-delivery, better mimics the natural cascade [34]. From a design standpoint, this specification requires hydrogels with erosion-controlled release mechanisms or affinity-based retention systems that maintain a constant release rate and protect growth factors from proteolytic degradation [14,32]. The recruitment of endogenous mesenchymal stem cells via sustained release of SDF-1α represents another key strategy during proliferation, requiring maintenance of chemokine bioactivity over the same extended timeframe [35,36].

### 2.3. Remodeling Phase: The Ultra-Sustained and Delayed-Start Mandate

The remodeling phase extends from approximately day 14 to months post-injury and is characterized by the replacement of granulation tissue with mature extracellular matrix, collagen cross-linking and re-organization, and myofibroblast apoptosis [6,37]. This stage requires precise regulation of the balance between matrix metalloproteinases (MMPs) and their endogenous inhibitors (TIMPs) to ensure proper ECM remodeling without excessive degradation [38].

The pharmacokinetic specification for hydrogel design is twofold: first, a delayed initiation of release (no significant release before day 10–14); second, ultra-sustained, low-dose release over weeks to months. Premature anti-fibrotic intervention impairs wound closure and increases dehiscence risk [39], while excessive or prolonged fibrosis leads to pathological scarring [38]. Therefore, anti-fibrotic agents—such as pirfenidone, TGF-β inhibitors, or FAK inhibitors—must be released only after the proliferative phase is complete [40,41]. This specification demands hydrogels with exceptionally slow degradation rates, typically achieved through high crosslinking densities or hydrophobic polymer backbones [27], combined with barrier layers or phase-delayed degradation mechanisms that create a lag period before payload release [42].

### 2.4. Transition Coordination: The Responsive-Release Mandate

The most challenging specification for hydrogel design is the precise coordination of transitions between phases. The inflammation-to-proliferation transition, governed by M1-to-M2 macrophage polarization, is particularly critical: premature transition risks unresolved infection, while delayed transition leads to chronic inflammation [6].

The pharmacokinetic specification is that release modulation should be triggered by microenvironmental cues rather than relying solely on preset time schedules. Biological markers such as MMP-9 upregulation, pH normalization, and glucose concentration changes serve as indicators of phase progression [16,43]. MMP-9, in particular, is elevated during the inflammatory phase and declines as healing progresses toward proliferation, making it a reliable indicator of the inflammation-to-proliferation transition [18]. Hydrogel systems must therefore incorporate environment-responsive elements—enzyme-cleavable peptide linkers, pH-labile bonds, or ROS-responsive crosslinks—that sense these cues and adjust release kinetics accordingly [17,44,45]. This “diagnostic” functionality enables the hydrogel to actively interrogate the wound microenvironment and release therapeutics only when the appropriate phase is detected, offering a level of precision that passive time-based release cannot achieve [21]. This concept of environment-responsive phase shifting directly establishes the design criteria for the responsive hydrogel systems detailed in Section 3.

Table 1 summarizes the pharmacokinetic specifications for each healing phase and the corresponding hydrogel design criteria that will be elaborated in Section 3.

## 3. Design Strategies for Programmable Release Kinetics

The preceding section established the biological rationale for phase-synchronized drug delivery: inflammation demands burst release, proliferation requires sustained zero-order kinetics, and remodeling calls for ultra-sustained, delayed-start release. Translating these pharmacokinetic specifications into tangible hydrogel systems requires deliberate engineering of the polymer network at multiple length scales—from molecular-level crosslinking chemistry to macroscopic architectural design.

This section examines the design strategies that enable programmable release kinetics. We begin with intrinsic network control—the passive programming of release through crosslinking density, degradation kinetics, and network architecture. We then introduce extrinsic, microenvironment-responsive mechanisms that enable hydrogels to sense and respond to phase-specific cues. Finally, we discuss how these strategies can be integrated within single systems to achieve the temporal precision required for therapeutic synchronization.

The polymers discussed throughout Section 3—natural, semi-synthetic, and synthetic—vary substantially in their source, biocompatibility, degradation behavior, and mechanical properties. Table 2 summarizes the comparative properties of the most commonly used hydrogel polymers in phase-synchronized delivery systems, providing a reference for material selection in subsequent design strategies.

### 3.1. Intrinsic Network Control: Passive Programming of Release Kinetics

Intrinsic network control refers to the design of hydrogel networks whose release kinetics are predetermined by their chemical and physical structure at the time of fabrication. These “passive” systems do not actively sense or respond to environmental changes; rather, their release profiles are encoded into the network architecture itself. While less adaptable than responsive systems, intrinsic control offers the advantages of simplicity, reproducibility, and predictable performance—qualities that have enabled the clinical translation of several hydrogel-based drug delivery platforms.

#### 3.1.1. Crosslinking Density as a Kinetic Governor

Crosslinking density is one of the most fundamental parameters governing release, operating alongside other factors such as drug–polymer affinity, electrostatic interactions, and hydrophobicity [53]. In covalently crosslinked hydrogels, crosslinking density determines the mesh size of the polymer network, which in turn controls the diffusivity of encapsulated cargo [14,15]. Tightly crosslinked networks (high crosslinking density) possess smaller mesh sizes, restricting the diffusion of macromolecular therapeutics and prolonging release duration. Conversely, loosely crosslinked networks facilitate faster diffusion and more rapid payload release [54,55]. This relationship between network structure and transport properties provides the foundation for tuning release kinetics across a wide range of hydrogel compositions [12,56]. It should be noted that crosslinking density is not the sole determinant of release kinetics. Drug–polymer affinity, electrostatic interactions, and hydrophobic effects can significantly influence partitioning and diffusional behavior, sometimes overriding the steric restrictions imposed by mesh size [53]. For charged or hydrophobic cargoes, these interactions may dominate release profiles regardless of crosslinking density. Therefore, crosslinking density modulation should be considered within the broader context of polymer–drug compatibility and network chemistry.

In disulfide-crosslinked PEG hydrogels, degradation time—and thus protein release kinetics—could be modulated from 2 days to 32 days by controlling crosslinking density through thiol precursor concentration [14]. In carboxymethyl chitosan-genipin hydrogels, increasing crosslinking density from 1% to 5% enabled precise modulation of drug delivery kinetics, with lower crosslinking density favoring enhanced diffusional mobility [54]. In chitosan-based microspheres, varying crosslinker concentration produced a wide range of release profiles attributable to differences in surface characteristics, porosity, and crosslinking density [57]. In freeze–thawed PVA hydrogels, the number of freeze–thaw cycles directly influences crystallite formation (physical crosslinks), thereby modulating network morphology and transport properties [58].

Crosslinking density can be modulated through several approaches: varying crosslinker concentration during synthesis [55], adjusting polymer concentration [13], controlling the degree of functionalization of polymer chains [59], or employing crosslinkers with different functionalities [60]. Each approach offers a different lever for tuning release kinetics while preserving other material properties.

Beyond diffusional control, crosslinking density also governs degradation-controlled release. In thiol-acrylate photopolymers, ester hydrolysis rates—and thus release profiles—are predictable from crosslinking density and buffer pH [61]. In photopolymerized PEG hydrogels for gene delivery, crosslinking density similarly controlled the release of plasmid DNA, enabling sustained transfection of encapsulated cells [62]. In hydrophobically modified alginate systems, drug release rates decreased as crosslinker density increased, with profiles fitting a Fickian relationship [60]. In dual-crosslinked gelatin/dextran hydrogels, the highest crosslinking density formulation exhibited sustained release for up to five weeks [63].

The underlying mechanism is well-established: higher crosslinking density reduces mesh size and increases diffusion pathway tortuosity [55]. This enables release profiles spanning hours to weeks by adjusting crosslinker concentration, polymer concentration, or crosslinker functionality [61,64], making crosslinking density one of the most widely applicable strategies for intrinsic control [12,59].

#### 3.1.2. Degradation-Controlled Release: Programming Release Through Network Disassembly

While crosslinking density governs diffusional release through mesh-size restriction, degradation-controlled release offers an orthogonal pathway for programming delivery kinetics. In degradable hydrogels, payload release is governed not by diffusion through a static network but by the progressive disintegration of the network itself [14,61]. The rate-limiting step shifts from solute diffusivity to the kinetics of network disassembly, enabling degradation-controlled systems to achieve near-zero-order release over extended periods—a profile difficult to realize with diffusion-controlled systems alone [65,66].

Hydrolytic degradation relies on the cleavage of ester, anhydride, or carbonate linkages, with degradation rates determined by polymer hydrophilicity, crosslinker structure, and crosslinking density. As crosslinking density decreases, the network becomes more accessible to water, accelerating hydrolysis and payload release [61]. This relationship enables degradation times ranging from days to months by adjusting crosslinking density or polymer composition [14]. The degradation–release coupling has been directly quantified in PLA-PEG-PLA hydrogels, where gel erosion correlated with a biphasic release profile: slow initial release followed by accelerated release as the network disassembled [67]. Thermo-responsive PLGA-PEG-PLGA hydrogels have further extended this platform to neuroprotective therapies, demonstrating the versatility of this degradation mechanism across different therapeutic contexts [68]. Supramolecular chitin-based hydrogels with reversible host–guest crosslinks achieve rapid degradation (~2 days) via enzymatic cleavage, eliminating the need for surgical removal and adapting to irregular wound geometries [69].

Enzymatic degradation offers localized, biologically triggered control over network disassembly. Gelatin methacryloyl (GelMA) hydrogels are susceptible to matrix metalloproteinase (MMP)-mediated degradation, with rates tunable by methacrylation degree [70]. Hydrogels crosslinked with MMP-9-cleavable peptides release payloads specifically in response to elevated MMP-9 concentrations in inflammatory wound microenvironments [18]. MMP-9 induces dose-dependent degradation, achieving >95% degradation within 14 days, enabling the hydrogel to sense wound state and release therapeutics only when appropriate [71,72].

Redox-responsive degradation harnesses disulfide bonds that cleave under reductive conditions characteristic of inflamed tissues. Beyond the classic PEG-dithiol system [14], recent advances have expanded this platform through light-triggered gelation of dynamic disulfide networks, enabling on-demand degradation and on-demand dissociation for wound repair [73]. Alternatively, disulfide-crosslinked hydrogels have been designed to respond to cell-secreted molecules, enabling autonomous, cell-controlled release [74].

Sequential degradation achieves multi-stage release through differential degradation rates. Intelligent sequential degradation hydrogels with concentric structures release an outer-layer antibacterial payload for early ROS scavenging, followed by an inner-layer regenerative payload for sustained immune modulation and angiogenesis [75,76]. The degradation mode—surface erosion (near-zero-order kinetics) versus bulk degradation (burst release)—further influences release profiles. In alginate/chitosan hydrogel coatings, this distinction was quantified through double or triple-phase zero-order release kinetics, demonstrating how erosion-controlled systems maintain network integrity and enable sustained release [2,65]. Bilayer hydrogels with distinct degradation rates in each layer enable cascade release synchronized with wound healing stages [42].

Because degradation-controlled release does not depend on cargo size, it is applicable to small molecules, proteins, and nanoparticles, making it a versatile strategy for sustained delivery [14,77].

**Figure 2:**
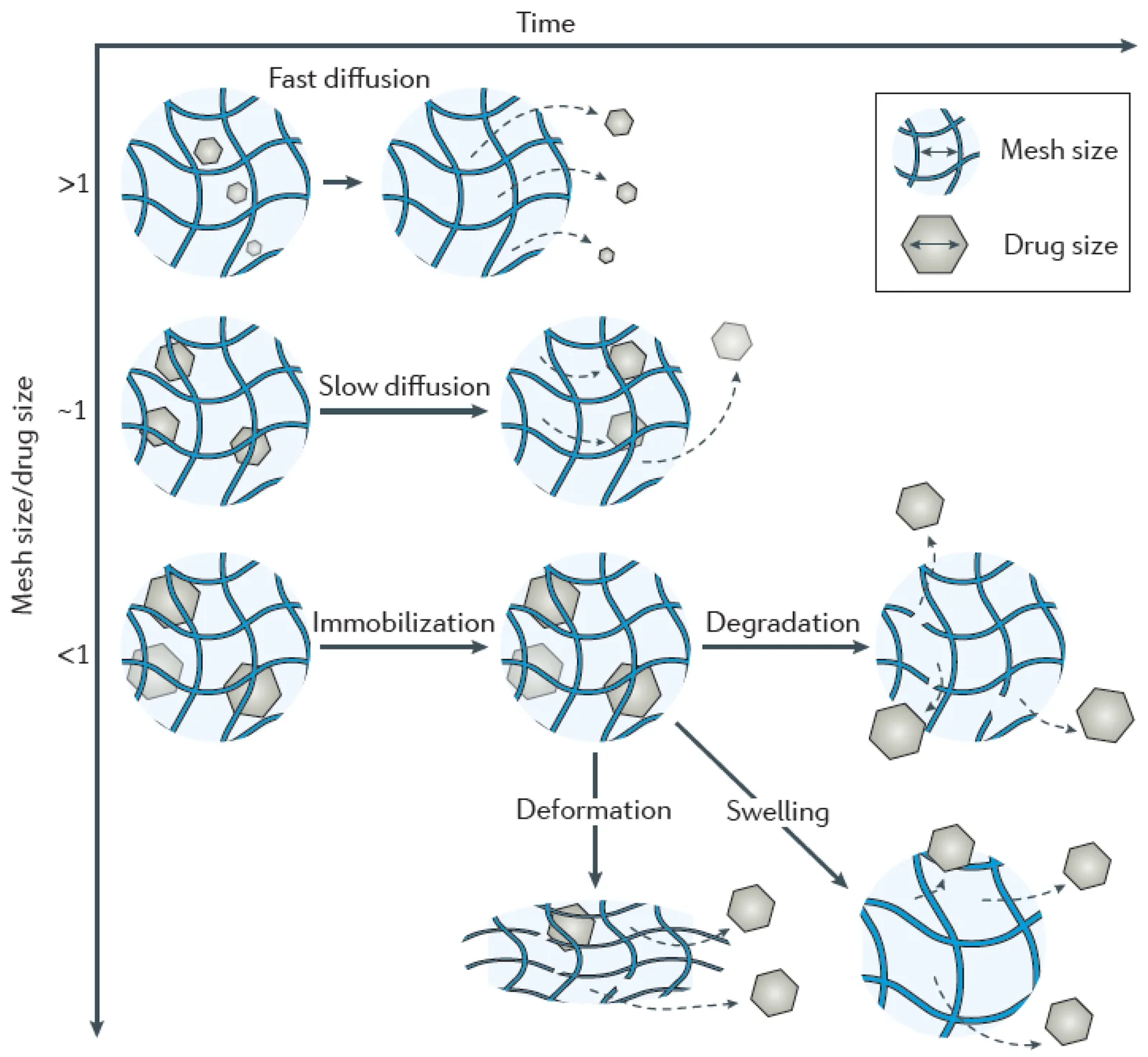
Intrinsic release mechanisms in hydrogel networks. The schematic illustrates how mesh size governs drug diffusion. A small drug relative to the mesh size diffuses rapidly, resulting in short release; when the drug size approaches the mesh size, release is dramatically slowed; when the drug is larger than the mesh size, it is physically entrapped. To release the immobilized drugs, the mesh size can be enlarged through network degradation, swelling, or mechanical deformation. Reproduced from Ref. [78].

The intrinsic release mechanisms discussed in Section 3.1.1 and Section 3.1.2—diffusion-controlled, degradation-controlled, and swelling-controlled release—are governed by the network mesh size and its dynamic changes. These mechanisms are schematically summarized in Figure 2.

#### 3.1.3. Architectural Engineering: Spatial Design for Programmed Release

While crosslinking density and degradation kinetics control release through molecular-level network properties, architectural engineering offers a complementary strategy at the microscale and macroscale. By spatially organizing the hydrogel into distinct structural domains—bilayers, core–shell constructs, Janus architectures, or graded structures—researchers can program release profiles that evolve over time and space, achieving sequential or staged delivery that aligns with the temporal progression of wound healing.

Bilayer and multilayer hydrogels represent the most straightforward architectural approach to sequential release. Zhang et al. developed a double-layer hydrogel system where the lower layer containing curcumin-loaded chitosan nanoparticles provides early anti-inflammatory and antioxidant effects, while the upper layer with pirfenidone-encapsulated gelatin microspheres delivers late-stage anti-fibrotic activity, with the varying degradation rates of each layer facilitating cascade release [42]. A pH-responsive bilayer hydrogel based on methacrylated hyaluronic acid (MeHA) layers with different degrees of methacrylation was designed: the lower layer (0.8% MeHA) degrades rapidly at acidic pH to release morin-based nanoparticles, while the upper layer (2% MeHA) degrades gradually at alkaline pH to sustain bFGF release [79]. A plaster-like bilayer hydrogel patch (NIM@UP/CuS@BT gel) features a glucose-responsive bottom layer containing copper sulfide (CuS) for transient antibacterial activity, and a stable upper layer incorporating nimesulide (NIM) for sustained anti-inflammatory effects [80].

Core–shell architectures offer a spatial design where the shell acts as a diffusion barrier or gatekeeper to control delivery timing. A 3D-printed chitosan/collagen scaffold with a pH-degradable polydopamine shell enables early release of antimicrobial peptides and antioxidant enzymes from the shell, while the core subsequently releases exosomes and EGF [81]. Core–shell bioactive glass nanoparticles sequentially deliver Cu^2+^ for early antibacterial action and Mg^2+^ for later tissue repair [82]. Core–shell hydrogel microspheres encapsulating aspirin in the shell and erythropoietin in the core provide rapid anti-inflammation followed by sustained osteogenic factor release [83].

Janus hydrogels—named after the two-faced Roman god—feature asymmetrical properties on opposing sides, enabling spatially segregated functionalities within a single construct [84]. A Janus-structured hydrogel integrated a thermostatic photothermal system with a temperature-responsive drug delivery system: the photothermal layer contains phase-change-regulated core–shell microspheres enabling temperature-triggered drug release, while the adhesive layer provides temperature-responsive shrinkage and controllable delivery [85]. An asymmetric double-layer Janus hydrogel was developed with programmed NO release, where high-concentration NO from the outer layer clears bacteria via photothermal action, while low and sustained NO from the inner layer (generated by glucose oxidase and L-arginine) exerts anti-inflammatory effects [84].

Gradient structures represent a more sophisticated architectural approach where crosslinking density, polymer composition, or drug loading varies continuously across the material. A stiffness gradient, achieved by modulating the concentration of methacrylated hyaluronic acid, facilitated skin cell organization in a hierarchically ordered configuration [86]. Gradient pH hydrogels have been shown to lower wound bed pH, reduce bacterial load, and induce macrophage polarization to M2 phenotype [87].

### 3.2. Extrinsic/Responsive Control: Active Programming of Release Kinetics

While intrinsic network control encodes release kinetics through passive structural features, active programming endows hydrogels with the ability to sense and respond to environmental cues, enabling on-demand release synchronized with dynamic healing processes. Stimuli-responsive hydrogels undergo reversible changes in polymer network structure upon exposure to triggers, broadly categorized as endogenous (pathological microenvironment) or exogenous (externally applied).

#### 3.2.1. Endogenous Triggers

pH-Responsive Systems. The pH of wounds varies substantially depending on wound type, healing stage, microbiological status, and patient-specific factors. Acute wounds typically exhibit a slightly acidic to neutral pH (approximately 5.5–7.0), while chronic or infected wounds often present elevated pH values (up to 8.0–9.0) due to bacterial metabolism and the breakdown of proteins into alkaline byproducts. These pH variations provide a potential trigger for pH-responsive hydrogel systems, though the specific pH range must be matched to the intended application [16,27]. A Salecan/hyaluronic acid hydrogel crosslinked by dynamic Schiff bases exhibited pH/hyaluronidase-dependent degradation and released polyhexamethylene biguanide specifically under acidic conditions, accelerating MRSA-infected wound healing [88]. Similarly, an alginate/graphene oxide dressing achieved accelerated levofloxacin release at pH 8.0 [89]. PβAE/PGS hydrogels increased release approximately 2-fold at pH 5.6 versus 7.4 [90], while Zn^2+^-coordinated carboxymethyl chitosan delivered ellagic acid for diabetic wounds in a pH-dependent manner [91].

ROS-Responsive Systems. Elevated ROS in inflamed tissues serves as another potent endogenous trigger. Thioketal-based hydrogels are widely employed: an injectable ROS/pH dual-responsive hydrogel crosslinked via a thioketal linker enabled co-delivery of therapeutics under oxidative/acidic conditions [92]; Exo-Gel incorporating thioketal moieties adaptively released stem cell exosomes in proportion to local ROS levels while scavenging excess ROS [93]. HTC/MCP hydrogels dissociate at high ROS to release Ca^2+^ and tannic acid, activating sustained growth factor release [94], while PACN@CG dual-network hydrogels encapsulating Cu/EGCG nanozymes enable cascade catalytic therapy in diabetic wounds [95].

MMP-Responsive Systems. Matrix metalloproteinases, upregulated during tissue remodeling, enable spatially controlled release precisely when matrix degradation occurs. MMP-responsive hydrogels have been reviewed for wound, bone, cartilage, myocardial, and neural repair applications [96]. A PEG-peptide hydrogel with MMP-cleavable crosslinks released isoquercitrin on-demand and promoted osteochondral defect repair [97]. The stage-matched conductive hydrogel PGO/CAM@Sal, incorporating pH/ROS/MMP9 triple-responsive salidroside release, suppressed pro-inflammatory cytokines and promoted angiogenesis in myocardial infarction models [98]. Additionally, Gel/GST-TIMP-bFGF hydrogels reduced ventricular wall thinning in infarcted rats [99], and gelatin/poly-L-lysine microneedles released curcumin upon MMP-triggered degradation [100].

Glucose-Responsive Systems. For diabetic wounds, glucose-responsive hydrogels offer adaptive release under hyperglycemia. A smart hydrogel using 4-carboxyphenylboronic acid-grafted ε-poly-L-lysine and oxidized konjac glucomannan loaded with magnolol and metformin exhibited glucose-responsive release and improved diabetic wound healing [101]. An injectable ODpCG-PB hydrogel functionalized with phenylboronic acid achieved shape-adaptive, glucose-responsive hemostasis [102]. Additional systems include AP/SA double-network hydrogels for adaptive insulin delivery [103] and glucose/pH dual-responsive ADSC-exosome releasing hydrogels acting through the Notch/NF-κB/NLRP3 pathway [104].

#### 3.2.2. Exogenous Triggers

NIR Light-Responsive Systems. Near-infrared light enables non-invasive, deep-tissue activation. A photothermally triggered bilayer pullulan/polydopamine hydrogel achieved 33% curcumin release under NIR versus 15% without NIR [105]. A lactoferrin@PNIPAM/SA/GO hydrogel reached a healing rate of 97.9% under NIR [106], and polydopamine carbon dot-based nanocomposite hydrogels achieved >99% bacterial eradication via NIR photothermal effect [107]. Additionally, pH/NIR dual-responsive chitosan/EGCG/hollow CuS hydrogels achieved 100% wound closure at day 13 in infected mice [108]. Despite its advantages, NIR-triggered release faces limitations in tissue penetration depth due to scattering and absorption by tissue components, with activation largely restricted to superficial or surgically accessible sites (typically 1–2 cm in soft tissues) [109]. Strategies such as upconversion nanoparticles or NIR-II agents are emerging to address this limitation but remain at early developmental stages [110].

Ultrasound-Responsive Systems. Ultrasound offers repeatable, non-invasive activation. An ultrasound-responsive hydrogel (URH) composed of alginate-thioketal-TiO_2_ and siRNA nanoparticles generated ROS under ultrasound to degrade the network and release siRNA, suppressing TGF-β1 in rat tendon injury models [37]. TT@P-Gel piezoelectric hydrogels combined electrical stimulation with controlled drug release, alleviating oxidative stress-induced nucleus pulposus senescence via AMPK-FOXO1a-mediated mitophagy [111]. CSGA-Cip composite hydrogels with dual dynamic crosslinks (Schiff base and Ag–S coordination) responded to low-frequency ultrasound in a graded manner: continuous ultrasound in the acute phase (0–3 d) accelerated Ag^+^ and ciprofloxacin release, while pulsed ultrasound in the repair phase (3–9 d) activated cell migration and angiogenesis [112]. PBE&SNO@HG, loaded with Prussian blue nanozymes and an ultrasound-responsive NO donor, achieved synergistic ROS scavenging and NO-driven angiogenesis in diabetic wounds [113]. Ultrasound offers deeper tissue penetration than NIR, with low-frequency ultrasound (20–100 kHz) reaching several centimeters, though high-frequency (>1 MHz) suffers attenuation in deeper tissues; propagation through bone or air-filled cavities is also limited [37].

Electro- and Magneto-Responsive Systems. Electrically conductive hydrogels respond to applied electric fields through reversible swelling or conductivity changes, suitable for electroactive tissues. Liquid metal microsphere-embedded conductive hydrogels achieved 99.1% wound closure in infected wounds via electrically triggered drug release [114], and hierarchically structured conductive patches enabled programmed release for diabetic wound healing [115]. Magnetic field-responsive systems, such as starch-g-oleic acid/gelatin/alginate magnetic core–shell microspheres, respond to alternating magnetic fields via heat generation or static field-induced deformation, offering additional spatiotemporal control for targeted delivery [116]. Electric fields face significant attenuation due to high impedance and insulating barriers such as the stratum corneum or bone, limiting penetration to superficial tissues unless invasive electrodes are used [114]. Magnetic fields offer the deepest tissue penetration among exogenous triggers, as they are largely unaffected by biological tissues, but require magnetic nanoparticles and specialized generators [117].

Beyond penetration depth, clinical feasibility of externally triggered systems faces barriers: NIR requires specialized light sources and often invasive fiber placement [118]; ultrasound demands precise transducer positioning and coupling [119]; electro-responsive systems need electrode implantation or skin patches [120]; magnetic systems require expensive generators [121]. All modalities face repeatability challenges—thermal damage, mechanical stress, or safety concerns—suggesting that translation requires user-friendly, deployable actuation technologies [122]. These considerations suggest that clinical translation requires not only material optimization but also user-friendly, clinically deployable actuation technologies [122].

**Figure 3:**
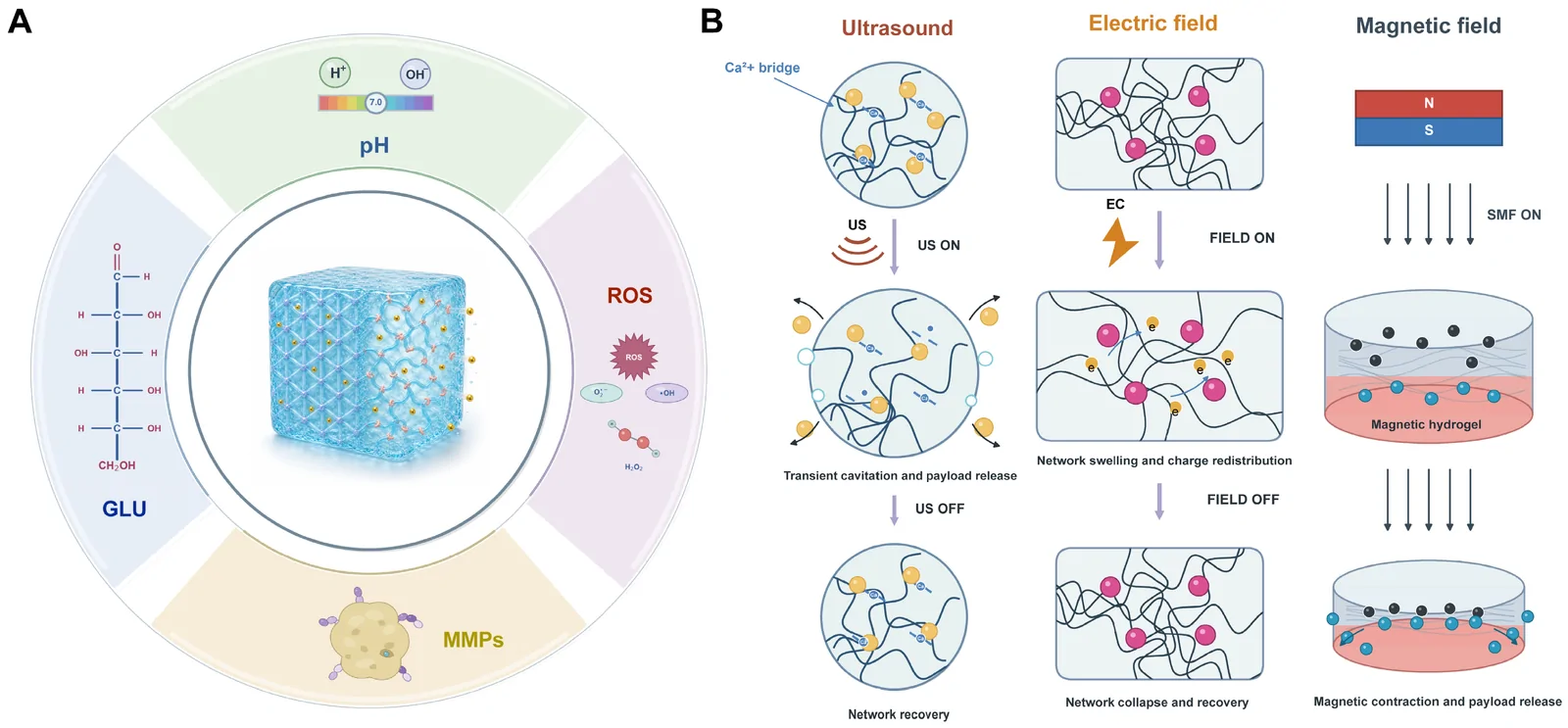
Stimuli-responsive mechanisms for active programming of release kinetics. (**A**) Endogenous triggers: Hydrogels incorporating pH-labile bonds (e.g., Schiff bases, acetals), ROS-cleavable moieties (e.g., thioketals, boronate esters), enzyme/MMP-cleavable peptide crosslinks, or glucose-responsive phenylboronate esters respond to pathological microenvironmental cues (acidic pH, elevated ROS, upregulated MMPs, and high glucose levels) through network degradation or structural reorganization, enabling on-demand drug release at the pathological site. (**B**) Exogenous triggers: Externally applied physical stimuli enable spatiotemporally controlled release. NIR light triggers photothermal effects and hydrogel contraction; ultrasound generates mechanical or thermal effects to modulate release; electric field induces reversible swelling changes in conductive hydrogels; magnetic field generates heat via magnetic nanoparticles or induces deformation.

Together, these endogenous and exogenous stimuli-responsive strategies provide a versatile toolbox for active programming of release kinetics, enabling hydrogels to adapt delivery precisely to the evolving demands of tissue repair. The mechanisms of the endogenous and exogenous triggers discussed above—including pH, ROS, MMP, glucose, NIR, ultrasound, electric, and magnetic stimuli—are schematically summarized in Figure 3.

### 3.3. Integrating Intrinsic and Extrinsic Control: Synergistic Design Paradigms

While Section 3.1 and Section 3.2 have discussed intrinsic (passive) and extrinsic (active) programming strategies separately, the most sophisticated hydrogel systems integrate both approaches within a single platform to achieve release kinetics that neither strategy could accomplish alone [123,124]. This integration creates synergistic design paradigms where passive architectural features—bilayer structures, core–shell geometries, or graded porosity—provide the foundational spatial compartmentalization, while responsive chemistries embedded within specific compartments enable environmentally triggered, stage-shift release. The combination addresses a fundamental limitation of purely passive systems (fixed release profiles that cannot adapt to dynamic pathological changes) and purely responsive systems (often lacking the structural hierarchy to achieve complex multi-stage delivery).

#### 3.3.1. Rationale for Integration

The biological rationale for integrating intrinsic and extrinsic control lies in the multi-phasic and spatially heterogeneous nature of tissue repair. Passive architectural features—such as a dense shell surrounding a porous core or a bilayer with distinct degradation rates—establish baseline release kinetics that align with the overall temporal progression of healing [123]. Superimposed on this baseline, responsive elements—pH-sensitive crosslinks that cleave in acidic wound environments, ROS-labile moieties that degrade under oxidative stress, or thermoresponsive polymers that undergo phase transition upon external heating—enable the system to accelerate, decelerate, or modulate release in response to real-time microenvironmental cues. This dual-layer control ensures that the material delivers therapeutics not only according to a pre-programmed schedule but also in direct proportion to the actual pathological state of the tissue.

#### 3.3.2. Synergistic Design Paradigms

Bilayer + Responsive Layer. One of the most widely explored synergistic designs combines bilayer architecture with stimulus-responsive chemistries in one or both layers. A plaster-like bilayer hydrogel patch (NIM@UP/CuS@BT gel) features a glucose-responsive bottom layer for transient antibacterial activity and a stable upper layer for sustained anti-inflammatory effects, achieving over 95% bactericidal efficacy [80]. A photo-controlled bilayer hydrogel enables light-assisted spatiotemporal sequential release of MXene–CeO_2_ nanozymes for photothermal sterilization and NO gas for angiogenesis, achieving a 97.74% wound closure rate [125]. The stage-matched conductive hydrogel (PGO/CAM@Sal) exemplifies a particularly elegant integration: its injectable conductive network provides structural support matching native myocardium, while pH/ROS/MMP9 triple-responsive moieties enable stage-specific salidroside release synchronized with myocardial infarction progression [98].

Core–Shell + Responsive Shell/Core. Core–shell architectures naturally lend themselves to synergistic integration, as the shell can be designed with passive barrier properties while simultaneously incorporating responsive elements. A thermoresponsive core–shell hydrogel for regenerative endodontics integrated chitosan–alginate microparticles for passive sustained release of growth factors with a thermosensitive matrix for temperature-triggered release, achieving sequential delivery of antibiotics and growth factors [126]. A 3D-printed core/shell fiber system with triple-drug loading achieved differential release rates from core, shell, and hollow channel compartments through combined passive structural control and active stimuli-responsive release [127]. A hydrogel embedded with core–shell bioactive glass nanoparticles enabled sequential release of Cu^2+^ for early antibacterial action and Mg^2+^ for later tissue repair [82].

Composite Hydrogels with Dual Crosslinking. Another powerful synergistic paradigm involves dual crosslinking mechanisms that combine intrinsic network control with environmental responsiveness. A carboxymethyl chitosan-based responsive dual-crosslinked hydrogel (CODEXD) utilized Schiff base crosslinks for pH-responsive diosmetin release at acidic infection sites, achieving high local drug concentrations for traumatic wound healing [128]. A dual-crosslinked hydrogel (AlgMA/GelMA) with time-sequential drug release was developed for stage-specific therapy of corneal alkali burns, enabling in situ formation and stage-matched pharmacotherapy [129]. This dual crosslinking strategy demonstrates how the integration of two crosslinking modes—one passive and one responsive—can achieve site-specific delivery.

#### 3.3.3. Design Considerations and Trade-Offs

Integrating intrinsic and extrinsic control introduces several design considerations that must be carefully weighed against the intended therapeutic application and the specific biological context.

Chemical compatibility. The passive and active components must be chemically compatible—responsive moieties (e.g., pH-labile Schiff bases, ROS-cleavable thioketals, or MMP-cleavable peptides) should not interfere with the formation or stability of the passive network structure. For instance, the introduction of labile crosslinks may reduce the mechanical integrity of the hydrogel, compromising its ability to serve as a structural scaffold. Similarly, the polymerization conditions required for the passive network (e.g., UV irradiation, temperature, or pH) must be compatible with the stability of the responsive moieties. This often requires iterative optimization of crosslinking conditions and monomer selection to achieve a balance between network stability and responsiveness [130].

Kinetic matching. The kinetics of the passive and active release mechanisms must be carefully synchronized. If the passive barrier degrades too quickly, the responsive element may be exposed prematurely, leading to a loss of phase-specific control; conversely, if the passive barrier degrades too slowly, the responsive element may be unable to modulate release in a timely manner. For example, in a bilayer system where the bottom layer is designed to be pH-responsive, the top (passive) layer must maintain its barrier function until the appropriate pH is encountered; premature top-layer degradation would compromise the entire release sequence. Achieving this kinetic match requires careful characterization of both degradation and drug-release kinetics under relevant physiological conditions [130].

Manufacturing complexity. The manufacturing complexity increases significantly with each additional level of integration, potentially affecting reproducibility, scalability, and cost. Systems that combine bilayer architecture, stimuli-responsive chemistry, and multiple therapeutic payloads require multi-step fabrication processes that are difficult to scale and validate under good manufacturing practice (GMP) conditions. Each additional processing step introduces potential sources of variability, from crosslinking efficiency to drug loading uniformity. This complexity also increases the cost of goods sold, which may limit the commercial viability of integrated systems compared to simpler passive formulations.

Programmability versus predictability. The trade-off between programmability and predictability must be managed carefully. While responsive systems offer greater adaptability to patient-specific microenvironments, their in vivo performance can be more variable due to patient-to-patient differences in pH, ROS, enzyme activity, and glucose levels. This variability makes it challenging to predict release profiles and therapeutic outcomes in individual patients. For systems designed for regulatory approval, the inherent variability of responsive systems may require larger clinical trials to demonstrate safety and efficacy across diverse patient populations. Passive systems, by contrast, offer more predictable and reproducible performance but lack the adaptive capabilities needed for personalized therapy. This trade-off must be weighed against the specific clinical application: for chronic, highly variable conditions (e.g., diabetic ulcers), the benefits of responsiveness may outweigh the additional uncertainty, whereas for more predictable healing contexts (e.g., clean surgical wounds), passive systems may be preferred [131].

Therapeutic cargo selection. The physicochemical properties of the therapeutic payload profoundly influence hydrogel design, stability requirements, and release strategies. Four major classes of cargo are commonly employed in phase-synchronized systems, each imposing distinct constraints on material selection.

Growth factors and proteins require protection from proteolysis and denaturation during fabrication and release. This necessitating affinity-based retention or carrier-assisted encapsulation strategies [132]. Exosomes demand preservation of membrane integrity and balanced porosity for spatiotemporally controlled release [133]. Nucleic acids require electrostatic complexation and protection from nuclease degradation, with stimuli-responsive release emerging as a promising platform [134]. By contrast, small-molecule drugs exhibit diverse physicochemical properties and primarily rely on diffusion governed by mesh size and drug-polymer affinity, imposing fewer stability constraints but requiring precise tuning of network structure [135,136,137].

The selection of therapeutic cargo thus dictates not only the release strategy but also the fundamental material choices—from polymer chemistry to crosslinking density to degradation mechanism. This interdependence must be considered early in the design process to avoid mismatches between cargo requirements and material properties.

Collectively, these trade-offs highlight that the design of integrated hydrogel systems is not a matter of simply combining passive and active features, but rather a process of deliberate optimization in which each design decision must be justified by the specific therapeutic context and translational pathway.

#### 3.3.4. Emerging Frontiers

Three levels of system sophistication can be distinguished in the context of responsive hydrogels. Stimuli-responsive materials represent the first level: they undergo structural or chemical changes upon exposure to a trigger (e.g., pH, ROS, or enzymes), but they do not actively sense, process information, or evaluate whether the therapeutic intervention has restored homeostasis [138]. Sensor–actuator systems represent the second level: they integrate a sensing element that detects a biological signal and a responsive element that releases drug accordingly, but the sensing and actuation are often decoupled, operate in an open-loop manner, or lack continuous feedback regulation [139,140]. True closed-loop platforms represent the third and most advanced level: they continuously monitor physiological biomarkers, process the information in real time, and autonomously adjust drug release to maintain therapeutic levels within a desired range, without external intervention [141,142].

The integration of intrinsic and extrinsic control is advancing toward closed-loop systems that sense and adapt in real time [143,144]. Biosensor-integrated hydrogels, AI-driven predictive release modeling, and 4D printing technologies represent the next frontier where passive architectural programming and active responsive control converge into truly intelligent therapeutic systems [130]. These advances promise to move the field from “passive drug depots” to “active therapeutic synchronizers”—materials whose chemical clock is set to the body’s biological clock.

Table 3 compares the three programming paradigms—intrinsic control, extrinsic/responsive control, and their integration—in terms of their mechanisms, advantages, and limitations.

The three programming paradigms differ substantially in temporal precision, adaptability, complexity, and translational potential. The choice among them must be guided by the therapeutic context and the required balance between programmability and predictability, as discussed in Section 3.3.3.

## 4. Tissue-Specific Applications of Phase-Synchronized Hydrogels

The design principles elaborated in Section 3—intrinsic network control, extrinsic responsiveness, and their integration—have been translated into therapeutic strategies across a diverse range of tissues. This section examines how phase-synchronized hydrogels have been applied to specific regenerative contexts, highlighting both commonalities in design logic and tissue-specific adaptations.

Achieving a desired release profile does not necessarily guarantee therapeutic success. The ultimate biological outcome depends on a complex cascade of events that extends beyond the temporal delivery of therapeutic agents: released molecules must engage target cells, activate specific signaling pathways, and orchestrate coordinated cellular responses—including migration, proliferation, differentiation, and immunomodulation—that collectively drive functional tissue regeneration [170,171]. Hydrogel properties such as stiffness, viscoelasticity, and ligand presentation directly influence cell behavior and signaling cascades, sometimes independently of the release kinetics they provide [172]. For instance, sustained growth factor release may be kinetically optimal, but if the hydrogel matrix fails to present appropriate biochemical cues or if the released factors lose bioactivity upon delivery, the therapeutic effect will be compromised. Conversely, a suboptimal release profile from a hydrogel that provides favorable mechanical cues and supports cell infiltration may still achieve acceptable biological outcomes. This distinction between release programming (the material’s ability to deliver cargo with precise timing) and therapeutic efficacy (the biological response to that delivery) is critical for interpreting preclinical results and guiding rational design [173]. The following sections evaluate representative applications with this distinction in mind, highlighting systems that have demonstrated not only controlled release but also meaningful biological outcomes.

### 4.1. Cutaneous Wounds and Diabetic Ulcers

Cutaneous wound healing proceeds through three overlapping phases: inflammation (hours to days), proliferation (days to weeks), and remodeling (weeks to months). Diabetic ulcers, characterized by persistent inflammation, elevated ROS, impaired angiogenesis, and delayed re-epithelialization, are a prime target for phase-synchronized hydrogel therapies.

Bilayer architectures achieve sequential release aligned with wound healing stages. A plaster-like bilayer hydrogel patch (NIM@UP/CuS@BT gel) achieved over 95% bactericidal efficacy through glucose-triggered sequential antibacterial and anti-inflammatory actions [80]. A programmable hierarchical dressing enables sequential PDGF-BB release for neutrophil recruitment and DNase I release for NETs degradation [174], while glucose/ROS/pH triple-responsive hydrogels promote macrophage polarization and accelerate healing [175]. In core–shell designs, a 3D-printed chitosan/collagen core–shell scaffold promoted M2 macrophage polarization and accelerated wound healing through programmed dual delivery [81].

### 4.2. Bone Defect Repair

Bone healing is characterized by an initial inflammatory phase, followed by angiogenesis and osteogenic differentiation, and finally bone remodeling. A spatiotemporally graded hydrogel system integrating a GelMA matrix (with DMOG) and silk fibroin microspheres (with nHAp) leverages differential degradation kinetics—rapid GelMA dissolution for prevascularization and sustained silk degradation for osteogenic maturation—achieving coordinated vascular network formation and mineralization [176].

Self-assembled hybrid hydrogel microspheres have also been designed to create a bone marrow-mimicking niche, enabling early BMSC/SKP release followed by OGP/KLT release for bone regeneration [177]. A CG@ZnO/BMP-2 hydrogel with rapid antibacterial and bone regeneration capabilities was developed via self-assembly, holding promise for bone infection treatment and enhancing bone healing while minimizing postoperative infection recurrence [178].

### 4.3. Cartilage and Tendon Repair

Cartilage and tendon repair present distinct challenges: avascular cartilage requires precise chondrogenic induction while suppressing fibrocartilage formation, whereas tendon healing demands coordination of inflammation regulation and tenogenic differentiation.

For cartilage regeneration, sequential delivery strategies have been explored through various systems: exosomal dual-drug hydrogels promoting BMSC recruitment followed by anti-fibrosis [179], time-programmed immunomodulatory agents followed by chondrogenic factors [180], and ROS-activated scaffolds enabling multi-stage anti-inflammation, chondrogenesis, and osteogenesis [181]. An aggrecanase-responsive hydrogel further enables miRNA-17 release based on inflammation levels [182]. For tendon repair, an inflammation-responsive core–shell micro-hydrogel enables SDF-1 release for MSC recruitment and IL-4 for M2 polarization [183], while a biphasic SIS/sodium alginate system provides sequential SDF-1α and BMP-12 release for stem cell recruitment and tenogenic differentiation, respectively [184].

### 4.4. Myocardial and Neural Regeneration

Myocardial and neural tissues present unique challenges due to their limited regenerative capacity and the need for precisely timed anti-apoptotic, pro-angiogenic, and anti-fibrotic interventions.

For myocardial infarction repair, bioactive cardiac ECM hydrogels achieve MMP-2-responsive sequential VEGF and Ang-1 release [185], while injectable deferoxamine/carvedilol-loaded hydrogels promote repair through anti-apoptosis and anti-inflammation [186]. ROS-differentiated Apelin-13 release systems reduce cardiomyocyte apoptosis within three days [187], and recombinant collagen-based hydrogels have shown efficacy in restoring cardiac function post-MI [188].

For neural regeneration, core–shell hydrogel microspheres with sequential delivery of cerium oxide nanoparticles and spinal white matter extracellular matrix have been developed for spinal cord injury, providing a biomimetic delivery strategy tailored to the dynamic pathological changes in SCI [189]. Controlled-release hybrid hydrogels delivering synergistic combinations of N-acetyl cysteine, ibuprofen, and progesterone achieve complete elution of NAC within 24 h, ibuprofen within 72 h, and progesterone over 21 days—timelines that correspond to the early antioxidative, anti-inflammatory, and neurotrophic phases of peripheral nerve repair [190]. A microenvironment-responsive chitosan-based injectable hydrogel with synergistic anti-inflammatory effects of triptolide and copper ions has been developed for spinal cord injury repair [191].

**Figure 4:**
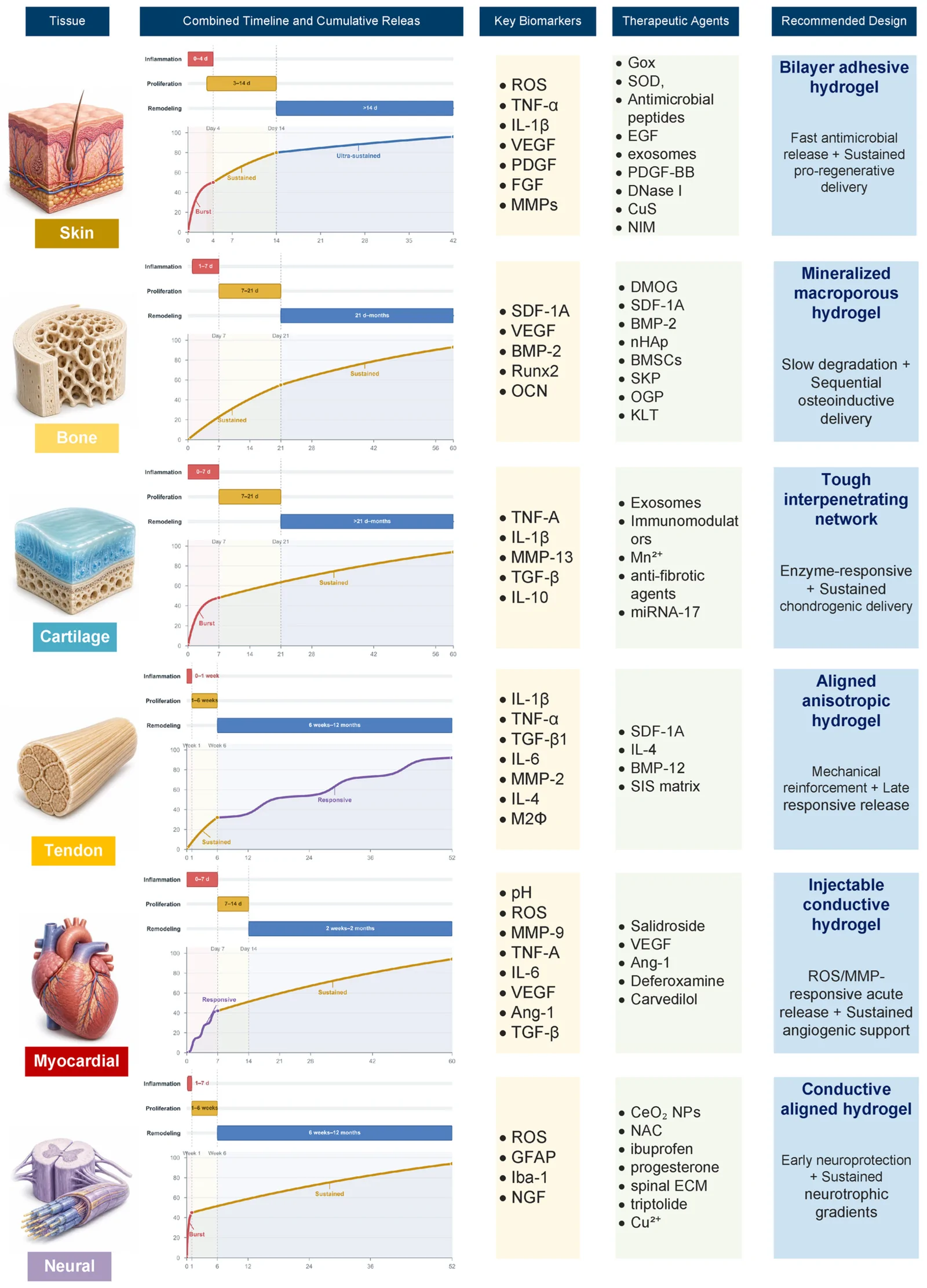
Decision-guided comparison of healing phase timings, biomarkers, therapeutic agents, and design strategies across tissue types. (**Left panel**): comparative healing timelines of skin, bone, cartilage/tendon, myocardial, and neural tissues with phase-specific release requirements. Colored bars denote inflammation (red), proliferation (yellow), and remodeling (green). (**Middle panel**): key biomarkers and representative therapeutic agents for each phase. (**Right panel**): recommended hydrogel design strategies, providing a practical reference for material selection.

The representative systems discussed above—along with additional examples from cutaneous, bone, and cartilage/tendon repair—are summarized in Table 4, and the healing timelines of the five tissue types are compared in Figure 4, providing an intuitive reference for cross-tissue comparison.

### 4.5. Commonalities and Divergences in Design Principles

Across these tissue-specific applications, several common design principles emerge. Construction of sequential-release hydrogels hinges on three factors: release mechanisms, spatial structure, and stimuli-responsiveness [22]. Bilayer and core–shell architectures provide spatial compartmentalization, while differential degradation kinetics (hydrolytic, enzymatic, or stimuli-responsive) program release timing [23]. Integration of passive architecture with responsive chemistries enables adaptation to wound pathology.

However, tissue-specific adaptations are equally evident. Wound healing hydrogels prioritize burst antimicrobial/antioxidant release followed by angiogenic signals, with glucose responsiveness particularly relevant for diabetic applications. Bone regeneration emphasizes angiogenesis–osteogenesis coordination over longer sustained windows [23]. Cartilage and tendon repair require immunomodulation followed by differentiation cues while avoiding fibrosis. Myocardial and neural applications focus on anti-apoptotic/anti-inflammatory action in the acute phase, transitioning to pro-angiogenic/regenerative signals later. These divergences underscore that while the design toolkit is broadly applicable, release timing must be tailored to each tissue’s healing dynamics. Importantly, these observations validate the phase-matching concept across diverse tissue contexts.

Direct quantitative comparisons between studies remain challenging due to heterogeneity in animal models (murine, rat, porcine, canine) that differ markedly from human physiology [192]. Injury models, treatment duration, outcome metrics, and evaluation timing are seldom standardized [193]. Rodent healing occurs primarily through contraction rather than re-epithelialization, limiting direct translatability [192], with rodent models achieving ~53% concordance with human outcomes versus 78% for porcine models [194]. These limitations underscore the need for caution in cross-study interpretation and increased use of large animal models.

## 5. Outlook, Challenges, and Concluding Remarks

While the design strategies and tissue-specific applications discussed above demonstrate remarkable progress, substantial hurdles remain in translating these phase-synchronized hydrogels and their precisely programmed release timelines [173]. Moreover, emerging technologies are poised to address current limitations and open new frontiers. This final section critically examines the key translational bottlenecks, highlights next-generation directions, and offers a concluding perspective on the evolution of hydrogels from passive depots to active therapeutic synchronizers.

### 5.1. Translational Bottlenecks

Despite the explosion of preclinical studies demonstrating sophisticated release kinetics, the clinical translation of phase-synchronized hydrogels remains limited [173].

In vivo–in vitro discrepancies represent a fundamental obstacle. While hydrogel degradation and drug release are routinely characterized in buffered solutions or simulated physiological media, the in vivo microenvironment is far more complex—characterized by dynamic fluid flow, protein adsorption, cellular infiltration, and enzymatic activity that can significantly alter degradation kinetics and release profiles. For instance, the concentration of MMPs in chronic wounds varies substantially across patients and over time, making the performance of MMP-responsive systems difficult to predict from in vitro data alone. This disconnect is particularly consequential for phase-synchronized systems, whose therapeutic efficacy depends on precisely timed transitions between release stages—transitions that may be delayed, accelerated, or obliterated in the unpredictable in vivo environment.

Cargo stability and bioactivity preservation pose additional challenges. Many therapeutic payloads—particularly growth factors, exosomes, and nucleic acids—are inherently unstable and susceptible to denaturation, aggregation, or proteolytic degradation during hydrogel fabrication, sterilization, and release. The encapsulation and delivery processes themselves can compromise bioactivity, even when release kinetics appear optimal. Strategies such as nanoparticle encapsulation, polyelectrolyte complexation, and cryoprotection have been explored, but a universal solution remains elusive.

Sterilization and scale-up represent practical but often overlooked barriers [195,196]. Standard sterilization methods—autoclaving, ethylene oxide treatment, and gamma irradiation—can degrade hydrogel networks, alter crosslinking density, or compromise stimuli-responsive moieties [195]. The development of sterilization-compatible materials and processes is therefore a prerequisite for clinical translation. Similarly, transitioning from laboratory-scale synthesis (milligrams to grams) to industrial-scale production (kilograms) introduces variability in crosslinking efficiency, drug loading uniformity, and batch-to-batch reproducibility that must be rigorously controlled to ensure that the programmed release kinetics are preserved across manufacturing batches. Beyond sterilization, economic and manufacturing scalability pose substantial hurdles for clinical translation [197]. The synthesis of stimuli-responsive polymers, incorporation of multiple therapeutic agents, and fabrication of complex architectures (bilayer, core–shell, or gradient structures) require multi-step processes that are difficult to scale while maintaining batch-to-batch consistency under Good Manufacturing Practice (GMP) conditions. The high water content of hydrogels further complicates storage, sterilization, and process optimization, requiring cost-intensive infrastructure. For clinical adoption, manufacturers must balance therapeutic performance against production costs, as integrated systems inherently carry higher manufacturing expenses than simpler passive formulations.

Regulatory pathways are complicated by the classification of phase-synchronized hydrogels as potential combination products—devices incorporating drugs or biologics [198]. In the United States, such products may fall under FDA’s Center for Devices (CDRH), Center for Drug Evaluation (CDER), or the Office of Combination Products (OCP), depending on the primary mode of action. In the European Union, the Medical Device Regulation (MDR, 2017/745) imposes rigorous requirements for clinical evaluation and post-market surveillance. The presence of stimuli-responsive crosslinkers and degradation byproducts further complicates safety assessments. Their long-term biocompatibility and immunogenicity profiles remain systematically unevaluated. To date, the majority of phase-synchronized hydrogel systems remain at the proof-of-concept stage in rodent models, with only a few having progressed to large animal studies, and none having entered human clinical trials.

Despite the progress reviewed above, several critical research gaps remain. First, real-time monitoring of drug release in vivo remains challenging, as most studies rely on endpoint measurements rather than continuous biosensing that could provide feedback on release timing. Second, few studies have rigorously demonstrated a direct causal link between the timing of drug release and biological phase transitions, such as M1-to-M2 macrophage polarization—most systems show temporal alignment but lack mechanistic evidence. Third, patient-specific variability in healing rates and microenvironmental conditions is rarely considered in current designs, limiting generalizability. Addressing these gaps will require interdisciplinary collaboration and a shift toward clinically meaningful endpoints.

**Figure 5:**
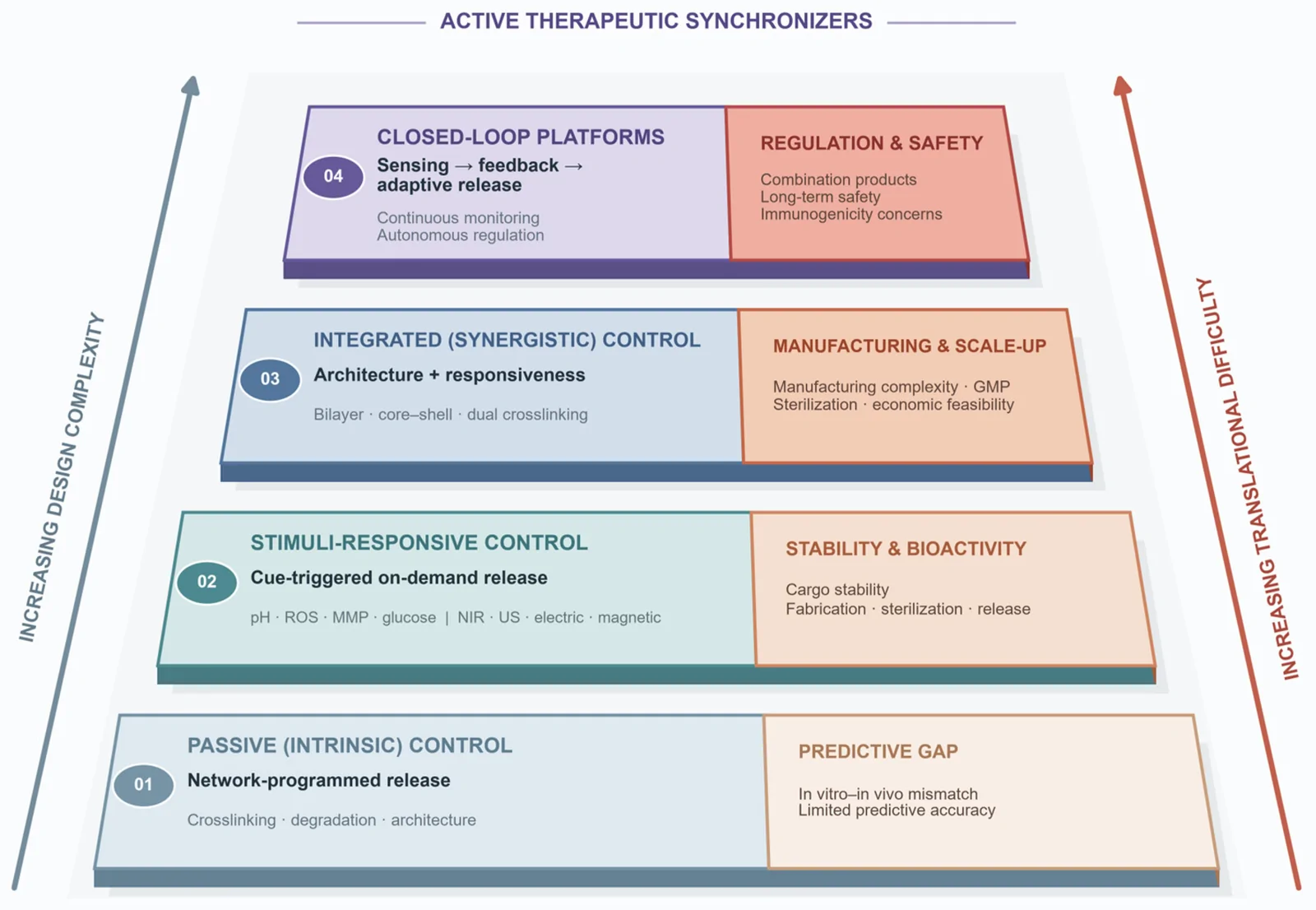
Progressive levels of release control and corresponding translational barriers. The left panel illustrates four hierarchical levels of hydrogel programming—passive (intrinsic) control, stimuli-responsive (active) control, integrated (synergistic) control, and closed-loop platforms—with increasing design complexity from bottom to top. The right panel summarizes the primary translational barriers associated with each level: in vitro–in vivo discrepancies, cargo stability and bioactivity preservation, manufacturing complexity and GMP scalability, and regulatory classification with long-term safety concerns.

The progressive levels of release control and their corresponding translational barriers are schematically summarized in Figure 5.

### 5.2. Next-Generation Frontiers

Addressing these bottlenecks and expanding the therapeutic potential of phase-synchronized hydrogels will require innovations that transcend current design paradigms. Three emerging frontiers are particularly promising.

As introduced in Section 3.3.4, closed-loop feedback systems represent the most advanced level of system sophistication. Recent proof-of-concept studies have demonstrated hydrogels coupled with electrochemical or optical sensors that detect wound infection markers and release antibiotics only when bacterial burden exceeds a threshold [199,200]. Such “smart” systems could adapt to patient-specific healing trajectories and dynamically modulate therapy as the wound progresses or regresses, effectively closing the loop between material programming and biological timing.

Artificial intelligence and machine learning are poised to accelerate hydrogel design and optimize release kinetics [201,202,203]. Predictive modeling approaches, including molecular dynamics simulations and machine learning algorithms, can screen vast libraries of polymer compositions, crosslinker chemistries, and drug combinations to predict release profiles without exhaustive experimental testing [201]. AI-driven design could optimize the timing and magnitude of sequential release, accounting for patient-specific variables such as wound size, location, and comorbidities.

4D printing and shape-memory mechanotherapies add the dimension of time-dependent structural evolution to hydrogel design [204,205,206]. 4D-printed hydrogels can undergo programmed shape transformations—such as folding, rolling, or swelling—in response to environmental stimuli, enabling dynamic mechanical cues that mimic the changing stiffness and geometry of healing tissues [205]. Shape-memory hydrogels that recover predefined shapes upon thermal or hydration stimuli could deliver therapeutic agents mechanically, in addition to chemically, and provide structural support that adapts to the wound bed.

### 5.3. Concluding Perspective

The evolution of hydrogel-based drug delivery systems from simple drug depots to sophisticated phase-synchronized therapeutic platforms reflects a broader paradigm shift in regenerative medicine: the recognition that effective healing requires not only the right therapeutic agents but also their delivery at the right time and in the right sequence. As this review has illustrated, the confluence of intrinsic network engineering—through crosslinking density, degradability, and architectural design—with extrinsic responsiveness to both endogenous and exogenous stimuli enables release kinetics that increasingly resemble the complex dynamics of natural tissue repair.

Based on the framework established in this review, we propose that a hydrogel system can be considered genuinely phase-synchronized when it meets the following criteria: (1) temporal precision—release kinetics are intentionally programmed to match the known temporal windows of specific healing phases; (2) multi-agent coordination—the release sequence follows the biological logic of the healing cascade (e.g., antimicrobial → angiogenic → anti-fibrotic); (3) biological feedback—the system incorporates responsiveness to phase-specific biomarkers (e.g., MMP-9, pH, ROS) that modulate release according to actual healing progression; (4) demonstrated phase-specific efficacy—the system promotes biological transitions between healing phases (e.g., M1-to-M2 macrophage polarization) through its release kinetics; and (5) tissue-specific adaptation—design parameters are tailored to the healing timeline and pathophysiological characteristics of the target tissue. Biological feedback responsiveness (e.g., to MMP-9, pH, or ROS) is an additional advantage for adaptive systems but is not mandatory for passive pre-programmed systems to be considered phase-synchronized. Systems meeting these criteria represent the current state-of-the-art, while future developments should aim to achieve fully closed-loop adaptation, autonomous biomarker sensing, and patient-specific programmability.

Yet the ultimate goal extends beyond matching release profiles to healing phases. It is the integration of materials science, biology, and engineering toward a unified vision: hydrogels as active therapeutic synchronizers that not only release drugs but also sense, adapt, and respond to the evolving needs of the patient. Achieving this vision will require continued interdisciplinary collaboration, rigorous translational research, and a willingness to embrace complexity. As the field matures from proof-of-concept to clinical reality, the promise of phase-synchronized hydrogels—materials whose chemical clock is set to the body’s biological clock—offers a transformative path forward for regenerative medicine.

While the concepts of “therapeutic synchronizers” and “chemical clocks” are intellectually compelling, the majority of evidence discussed in this review derives from preclinical studies, predominantly in rodent models. The transition from proof-of-concept to clinical reality entails substantial challenges—including scalability, regulatory approval, long-term safety, and patient-specific variability. The promising results reported to date should therefore be interpreted as indicators of potential rather than established clinical efficacy. Rigorous validation in large animal models, followed by well-designed human trials, remains essential before phase-synchronized hydrogels can be considered clinically ready. With these caveats in mind, the field holds genuine promise for advancing regenerative medicine.

## Figures and Tables

**Table 1:** Healing Phase-Specific Pharmacokinetic Specifications and Hydrogel Design Criteria.

Healing Phase	Timeframe	Key Biological Events	Pharmacokinetic Requirement	Desired Release Profile	Key Design Strategy	Ref.
Inflammatory	0–4 days	Neutrophil infiltration, ROS burst, M1 macrophage dominance	Rapid, high-concentration antimicrobial/antioxidant delivery	Burst release (hours–2 days)	Low crosslinking density, highly porous architecture, pH/ROS-labile crosslinks	[15,16,17,28]
Proliferative	3–14 days	Fibroblast/EC proliferation, angiogenesis, granulation tissue formation	Sustained growth factor signaling (VEGF, PDGF, FGF, SDF-1α)	Sustained/zero-order release (7–14 days)	Erosion-controlled networks, affinity-based retention, degradation-controlled release	[14,32,34,36]
Remodeling	>14 days	Collagen cross-linking, ECM maturation, myofibroblast apoptosis	Delayed-start, prolonged anti-fibrotic signaling	Ultra-sustained, delayed-start release (weeks–months)	High crosslinking density, barrier layers, phase-delayed degradation	[27,38,40,42]
Phase Transition	Variable	M1 → M2 macrophage polarization, MMP upregulation, pH normalization	Environment-triggered release modulation	On-demand/adaptive release	MMP-cleavable peptides, pH-labile bonds, ROS-responsive crosslinks	[15,18,43,44]

**Table 2:** Comparative Properties of Common Hydrogel Polymers.

Polymer	Source	Biocompatibility	Degradation Time	Mechanical Strength	Key Advantages	Limitations	Ref.
Chitosan	Natural (crustacean)	Excellent	Days–weeks (enzymatic)	Low–Moderate	Antibacterial activity; antioxidant; cationic; structurally tunable	Low mechanical strength; rapid degradation; poor water solubility	[46]
Hyaluronic acid (HA)	Natural (animal)	Excellent	Days–weeks (enzymatic)	Low	Highly biocompatible; FDA-approved; cell adhesion properties	Poor printability; insufficient mechanical properties; uncontrolled degradation	[47]
Gelatin (GelMA)	Natural (collagen derivative)	Excellent	Weeks (enzymatic, tunable)	Low–Moderate (10–60 kPa)	Cell-adhesive; MMP-sensitive; photocrosslinkable; tunable stiffness	Moderate mechanical properties; batch variability	[48]
Alginate	Natural (algae)	Good	Weeks–months (ion leaching)	Moderate	Inexpensive; injectable (Ca^2+^ crosslinking); gel-forming	Limited cell adhesion; non-degradable in mammals	[49]
PEG	Synthetic	Excellent	Non-degradable (unless modified)	Moderate–High	Highly tunable; low protein adsorption; FDA-approved; soft-tissue-like mechanics	Biologically inert; non-degradable unless modified	[50]
PLGA	Synthetic	Good	Weeks–months (hydrolytic, 40–84 days)	High	FDA-approved; controllable degradation; non-toxic; non-immunogenic	Acidic byproducts; hydrophobic	[50]
PNIPAM	Synthetic	Good	Weeks–months (non-degradable unless modified)	Moderate (40–280 kPa)	Thermo-responsive (LCST ~32 °C); injectable; volume phase transition	Non-degradable; dehydration/volume change during phase transition	[51]
PVA	Synthetic	Good	Weeks–months (physically crosslinked)	Moderate–High (18–200 MPa)	Mechanical strength; freeze–thaw crosslinking; tunable properties	Non-degradable (some forms); property changes over time	[52]

**Table 3:** Comparison of Programming Paradigms for Phase-Synchronized Release.

Programming Paradigm	Mechanism	Key Design Parameters	Advantages	Limitations	Representative Examples
Intrinsic (Passive) Control	Release kinetics predetermined by network structure at fabrication	Crosslinking density, degradation kinetics, bilayer/core–shell/Janus/gradient architecture	Simple, reproducible, predictable performance; clinically translatable	Fixed profile cannot adapt to dynamic pathological changes	Crosslinking density modulation [14,54,55]; bilayer hydrogels [42,79]; core–shell [81,82,83,145,146,147]; Janus [84,85,148]
Extrinsic (Active) Control	Release triggered by endogenous or exogenous stimuli	pH-, ROS-, MMP-, glucose-responsive bonds; NIR, ultrasound, electro, magnetic responsiveness	On-demand, spatiotemporally precise; adapts to microenvironment	In vivo variability; patient-to-patient differences; external equipment dependence	pH-responsive [88,89,90,91,149,150,151,152,153,154]; ROS-responsive [92,93,94,95,155,156,157]; MMP-responsive [96,97,98,99,100,158,159,160,161]; glucose-responsive [101,102,103,104,162,163,164]; NIR [105,106,107,108,165,166]; ultrasound [37,111,112,113]
Integrated (Synergistic) Control	Passive architecture + responsive chemistry in single system	Bilayer + responsive layer; core–shell + responsive shell/core; dual crosslinking	Combines spatial compartmentalization with environmental adaptability	Manufacturing complexity; compatibility between passive and active components	Bilayer + responsive [80,125,167]; core–shell + responsive [126,127,168]; dual crosslinking [128,129,169]

**Table 4:** Representative Phase-Synchronized Hydrogel Systems Across Tissue Types.

Tissue/Application	Hydrogel System	Architecture/Design	First Phase (Early Release)	Second Phase (Late Release)	Release Profile Type	Release Duration	Biomarker/Biological Signal	Key Outcome	Ref.
Diabetic wound	3D-printed chitosan/collagen scaffold	Core–shell + pH-degradable shell	Antimicrobial peptides, antioxidant enzymes (shell)	Exosomes, EGF (core)	Burst → Sustained	0–3 d/3–14 d	pH 5.5–6.5, ROS	M2 polarization, accelerated healing	[81]
Infectious wound	Bioactive glass nanoparticle hydrogel	Core–shell nanoparticles	Cu^2+^: antibacterial, pro-inflammatory	Mg^2+^: tissue repair, anti-inflammatory	Burst → Sustained	0–3 d/3–14 d	pH, ROS, TNF-α	Stage-matched wound healing	[82]
Bone defect	Spatiotemporally graded hydrogel (GelMA + silk fibroin microspheres)	Graded structure + differential degradation	DMOG: prevascular network formation (rapid GelMA degradation)	nHAp: osteogenic maturation (sustained silk degradation)	Sustained → Sustained	21 days	VEGF, BMP-2, Runx2	Vascularized bone regeneration	[176]
Bone defect	Self-assembled hybrid hydrogel microspheres	BM-mimicking niche	BMSCs, SKP (early)	OGP, KLT (late)	Sustained → Sustained	0–10 d/sustained thereafter	SDF-1α, BMP-2	Critical bone defect repair	[177]
Cartilage	Exosomal dual-drug hydrogel	Sequential release system	BMSC recruitment, chondrogenesis	Inflammation suppression, anti-fibrosis	Burst → Sustained	24 h/30 days	TGF-β, IL-10, MMP-13	Hyaline cartilage repair	[179]
Tendon	Core–shell micro-hydrogel	Core–shell + inflammation-responsive	SDF-1: MSC recruitment	IL-4: M2 macrophage polarization	Sustained → Responsive	0–3 d/3–10 d	MMP-2, IL-4, M2 macrophages	Tendon-to-bone healing	[183]
Myocardial infarction	PGO/CAM@Sal conductive hydrogel	Injectable + pH/ROS/MMP9 triple-responsive	Salidroside: anti-apoptosis, anti-inflammation (acute phase)	Pro-angiogenic, regenerative (subacute/chronic)	Responsive → Sustained	0–7 d/7–21 d	pH, ROS, MMP-9, TNF-α, IL-6	Post-infarction myocardial repair	[98]
Spinal cord injury	Core–shell hydrogel microspheres	Core–shell + sequential delivery	Cerium oxide nanoparticles: ROS scavenging (first week)	Spinal white matter ECM: regeneration	Burst → Sustained	1 week/8 weeks	ROS, GFAP, Iba-1	Functional recovery in SCI	[189]

## Data Availability

No new data were created or analyzed in this study. Data sharing is not applicable to this article.

## Abbreviations

The following abbreviations are used in this manuscript:

BMP	Bone morphogenetic protein
BMSCs	Bone marrow mesenchymal stem cells
CC BY	Creative Commons Attribution
CO	Carbon monoxide
CuS	Copper sulfide
DMOG	Dimethyloxalylglycine
ECM	Extracellular matrix
EGF	Epidermal growth factor
FGF	Fibroblast growth factor
GelMA	Gelatin methacryloyl
GOx	Glucose oxidase
HIF-1α	Hypoxia-inducible factor-1α
IL	Interleukin
MeHA	Methacrylated hyaluronic acid
MI	Myocardial infarction
MMP	Matrix metalloproteinase
MRSA	Methicillin-resistant *Staphylococcus aureus*
NGF	Nerve growth factor
NIR	Near-infrared
NO	Nitric oxide
PDGF	Platelet-derived growth factor
PEG	Poly(ethylene glycol)
PNIPAM	Poly(N-isopropylacrylamide)
ROS	Reactive oxygen species
SA	Sodium alginate
SCI	Spinal cord injury
SDF-1α	Stromal cell-derived factor-1α
SIS	Small intestinal submucosa
TGF-β	Transforming growth factor-beta
TNF-α	Tumor necrosis factor-alpha
V EGF	Vascular endothelial growth factor
